# Protein Nutritional Status and Frailty: A Mendelian Randomization Study

**DOI:** 10.1093/jn/nxab348

**Published:** 2021-11-23

**Authors:** Yasutake Tomata, Yunzhang Wang, Sara Hägg, Juulia Jylhävä

**Affiliations:** Department of Medical Epidemiology and Biostatistics, Karolinska Institute, Stockholm, Sweden; School of Nutrition and Dietetics, Faculty of Health and Social Services, Kanagawa University of Human Services, Yokosuka, Kanagawa, Japan; Department of Medical Epidemiology and Biostatistics, Karolinska Institute, Stockholm, Sweden; Department of Medical Epidemiology and Biostatistics, Karolinska Institute, Stockholm, Sweden; Department of Medical Epidemiology and Biostatistics, Karolinska Institute, Stockholm, Sweden; Faculty of Social Sciences (Health Sciences) and Gerontology Research Center (GEREC), University of Tampere, Tampere, Finland

**Keywords:** Protein nutritional status, serum albumin, serum total protein, frailty, aging, Mendelian randomization

## Abstract

**Background:**

Observational studies have suggested that better protein nutritional status may contribute to prevention of frailty.

**Objective:**

We sought to examine this hypothesis using a Mendelian randomization (MR) analysis.

**Methods:**

We conducted a two-sample MR study using GWAS summary statistics data of the UK Biobank. We applied genetically predicted serum albumin as a primary exposure measure and serum total protein as a secondary exposure measure. The outcome measure was the Rockwood frailty index (FI) based on 49 deficits from 356,432 individuals (53.3% of them were women, with a mean ± SD age of 56.7 ± 8.0 y. The association between serum protein measures and FI was mainly analyzed by use of the inverse variance weighted method.

**Results:**

A genetically predicted serum albumin concentration was not statistically significantly associated with FI in the full sample. However, in women, we observed a preventive association between genetically predicted serum albumin and FI (β = −0.172 per g/L; 95% CI: −0.336, −0.007; *P *= 0.041). In the full sample, genetically predicted serum total protein was inversely associated with FI (β: −0.153 per g/L; 95% CI: −0.251, −0.056; *P *= 0.002). In both women and men, higher serum total protein was significantly inversely associated with FI; regression coefficients were −0.148 per g/L (95% CI: −0.287, −0.009; *P *= 0.037) for women, −0.154 per g/L (95% CI: −0.290, −0.018; *P *= 0.027) for men.

**Conclusions:**

The present MR study implies that better protein nutritional status modestly contributes to reducing the risk of frailty.

## Introduction

With a rapidly aging global population, frailty has become a major health issue (1, 2). Frailty is a geriatric syndrome that predicts functional disability and mortality. Being a predicable condition, frailty is reversible at least to some extent (1–4). Therefore, there is an urgent need to find preventive strategies to mitigate frailty in the aging society.

Although the prevalence of frailty is higher in older persons (5, 6), frailty is a consequence of cumulative decline in physiological systems during a lifetime (1). Hence, frailty is a research topic that does not affect just older adults. Previous cohort studies have reported that frailty is associated with a higher risk of mortality even in adults aged <50 y (5, 6). Therefore, a life-course perspective for frailty prevention is needed. Furthermore, there is a well-known sex difference in frailty. Women tend to be more frail than men (as indicated by a higher average frailty index level) although they have lower mortality rates (7). Recently, sex-specific strategies for the prevention of frailty were conceptually proposed based on sex differences in frailty traits (e.g., sarcopenia, osteoarthritis, depression, or cognitive function) although preventive sex-specific studies on frailty itself are still missing (8).

Protein nutritional status is considered a preventive factor for frailty (9, 10). To investigate the relation between protein intake and frailty status, a previous study has conducted a meta-analysis of observational studies that included a total of 50,284 older adults (age ≥60 y) from 7 cross-sectional and 3 longitudinal studies (frailty status was assessed by use of the Cardiovascular Health Study Frailty Index or the Kihon checklist), and the researchers concluded that higher consumption of dietary protein was inversely associated with frailty (11). For example, adequate protein intake is advocated as a possible intervention for the management of frailty in older adults due to its effects on muscle mass and physical function (10, 11). Serum albumin is a common blood measure to assess protein nutritional status in clinically stable individuals (12) as albumin is the most abundant protein in human serum (13). Serum albumin concentrations can be raised by nutritional supplementation (14, 15). In humans, the albumin turnover time of about 25 d normally reflects a liver albumin synthesis rate of about 10.5 g/d (16). Because serum proteins are mostly synthesized in the liver, not only poor protein and energy intake, but also impaired liver synthetic function can result in low circulating concentrations of serum proteins (13). Lower albumin concentration is associated with higher risks of mortality (12, 17), loss of muscle mass (12), and cardiovascular events (18). In clinical settings in postoperative patients, lower albumin concentration is a prognostic factor for delayed recovery and complications (e.g., infection) (19, 20). Furthermore, previous studies have suggested that dietary protein intake may have multiple preventive roles in several traits associated with frailty, such as cognition, mood, and comorbidity (e.g., bone health) (21). Maintaining better protein nutritional status across life may be important to prevent frailty.

Observational studies are generally susceptible to residual confounding and reverse causation. Indeed, a systematic review highlighted the influence of confounders on the association between protein intake and frailty (11). Therefore, to avoid residual confounding, a randomized controlled trial (RCT) is the best way to examine the causal relation between protein nutritional status and frailty. Several RCTs have examined the combined effect of protein supplementation and muscle strength training on frailty. A systematic review of intervention studies has concluded that “a combination of muscle strength training and protein supplementation was the most effective intervention to delay or reverse frailty” (3). In addition, an RCT demonstrated that protein supplementation alone improves muscle mass and physical performance in undernourished frail older adults (22). However, to the best of our knowledge, no RCT has yet reported results on the effect of single-component interventions for protein nutritional status on frailty.

To overcome the problem of confounding and reverse causation in observational studies, the Mendelian randomization (MR) approach is proposed (23). MR is a part of instrumental variable analyses, applying genetic variants [single nucleotide polymorphisms (SNPs)] as instrumental variables for the exposure of interest (23). MR is often described as a “natural RCT” because the random allocation of alleles during meiosis is conceptually similar to the random allocation of intervention in the RCT. Hence, the MR analysis can provide more robust evidence regarding the causal relation between serum proteins and frailty than observational studies.

Moreover, the MR study design examines the association of lifelong exposure because the genetic variants influence traits across the whole life course (24). However, as far as we are aware, no MR study has yet investigated the association between protein nutritional status and frailty, which is thus the aim of our study.

## Methods

### Study design

We conducted two-sample MR analyses using summary statistics of a genome-wide association study (GWAS). As an exposure measure of genetically predicted protein nutritional status by using established SNPs in the previous GWAS analysis, we applied serum albumin as a primary exposure measure and serum total protein as a secondary exposure measure. Serum albumin and serum total protein are widely used as indicators of protein nutritional status (12, 14), and serum albumin concentrations can be raised by nutritional supplementation (14, 15). The Rockwood frailty index (FI) (please see details under “Frailty”) was used as an outcome measure of frailty (25).

### Data sources

The primary analysis was based on data from the UK Biobank (26). The UK Biobank is a population-based cohort study on individuals aged from 40–69 y, recruited from the UK National Health Service registers. The UK Biobank includes data on genetic and environmental factors across the UK, collected cross-sectionally from 2006–2010 (https://www.ukbiobank.ac.uk/).

For data on the association between genetic variants and serum proteins, we used GWAS summary statistics from the UK Biobank (regression coefficients for serum proteins in g/L) released by the Neale Lab (https://github.com/Nealelab/UK_Biobank_GWAS). For a quality control of the samples, the GWAS summary statistics were considered using the following filter parameters: a principal components analysis calculation filter for selection of unrelated samples, a sex chromosome filter for removal of aneuploidy, a filter of principal components for European sample selection to determine British ancestry; and filters for selection of self-reported “white British”, “Irish”, and “white”. This GWAS included 315,268 individuals (white-British ancestry). We obtained 3 kinds of summary statistics data, for the full sample including both sexes (*n* = 315,268), women (*n* = 168,146, 53.3%), and men (*n* = 147,122, 46.7%). The details can be found at https://github.com/Nealelab/UK_Biobank_GWAS.

For the sensitivity analysis, we also used summary statistics data from the European-ancestry meta-analysis consisting of 53,190 individuals (from 20 GWASs) for serum albumin and 25,539 individuals (from 6 GWASs) for total protein (27).

For estimating the genotype-FI associations, we analyzed individual-level data from participants enrolled in the UK Biobank. Among the 500,336 participants who had the information about FI available (25), we included 356,432 participants who were *1*) genotyped, *2*) of European ancestry, *3*) had complete information on variables included in the FI, and *4*) not randomly dropped with a kinship threshold of 0.1768 for considering relatedness (**Supplemental Figure 1**).

### Selection of instrumental variables

As instrumental variables, we selected 6 SNPs for serum albumin and 2 SNPs for serum total protein (**Table 1**) that were significantly associated with serum proteins concentrations (*P* < 5 × 10^–8^) in the previous GWAS meta-analysis in individuals of European ancestry (27). The detailed information of the study design, phenotype definition, quality control, and imputation of the genetic data were described previously (27). In addition, all SNPs were significantly (*P* < 5 × 10^–8^) associated with serum proteins concentrations in the UK Biobank study (Table 1).

**TABLE 1:** Summary statistics of serum proteins-raising genetic variants^1^

				Serum protein markers^2^				Frailty index^3^		
SNP	Chr	Effect allele	Other allele	EAF	*β*	SE	*P* value	*β*	SE	*P* value
Serum albumin										
rs4806073	19	C	T	0.07	0.280	0.013	8.08 × 10^–106^	−0.006	0.034	0.873
rs1260326	2	T	C	0.61	0.153	0.007	5.02 × 10^–120^	0.040	0.018	0.023
rs11078597	17	C	T	0.19	0.170	0.008	3.34 × 10^–9^^4^	−0.007	0.022	0.757
rs13381710	18	G	A	0.30	0.066	0.007	6.28 × 10^–21^	−0.020	0.019	0.295
rs16948098	15	A	G	0.04	0.215	0.016	2.02 × 10^–39^	−0.082	0.043	0.060
rs739347	19	T	C	0.90	0.188	0.011	5.43 × 10^–69^	−0.042	0.029	0.141
Serum total protein										
rs3751991	17	A	C	0.10	0.427	0.017	1.18 × 10^–142^	−0.038	0.029	0.182
rs204999	6	A	G	0.69	0.250	0.011	1.18 × 10^–114^	−0.058	0.019	0.002

1 Chr, chromosome; EAF, effect allele frequency; SNP, single-nucleotide polymorphism.

2 Summary statistics for serum protein markers (albumin, total protein; g/L) from UK Biobank data.

3 Summary statistics for frailty index from UK Biobank data.

We also used another set of SNPs (6 SNPs for serum albumin, 3 SNPs for serum total protein) for serum proteins that were defined as “established loci” in the transethnic meta-analysis of European ancestry and Japanese GWASs (27). These SNPs have been used as instrumental variables in a previous MR study (28).

### Frailty

The Rockwood FI, based on the accumulation of deficits model, was used as an outcome measure of frailty. The FI is a continuous measure that also has high sensitivity at the lower end of the frailty continuum. According to the principles of this model (29), the FI value for each individual was calculated as the number of deficits present divided by the total number of 49 deficits as described in a previous study (25). In the present study, the FI value was expressed as a percentage (%). For example, an individual having 10 deficits has an FI of 10/49 = 0.204 (20.4%).

### Statistical analysis

We estimated the association of each genetic variant with the continuous FI using linear regression analyses and assumed an additive model to obtain summary statistics for the two-sample MR analyses. All the regression analyses were adjusted for age (continuous variable) and sex by using SAS version 9.4 (SAS Inc.). To obtain sex-specific summary statistics of frailty for sex-stratified MR analyses, we also conducted linear regression analyses stratified by sex (adjusted for age). We also conducted stratified analyses by age (<60 y or ≥60 y) and menopausal status (nonmenopausal, postmenopausal. *n* = 160,666 women) for the other stratified MR analyses.

Two-sample MR analyses were performed to calculate the coefficient (linear association) and 95% CIs for FI. All MR analyses were performed by using the “mrrobust” package in STATA version 15 (StataCorp LLC) and the “MendelianRandomization” package for R version 3.4.3 (The R Foundation for Statistical Computing). We primarily used the inverse variance weighted method with fixed effect standard errors (30). We also conducted additional sensitivity analyses with the inverse-variance weighted method with penalized weights and the MR-Lasso method to assess the influences of variants with outlying causal estimates (31). The inverse variance weighted method assumes that all variants are valid instrumental variables. In addition, we also conducted sensitivity analyses using the weighted median method and the MR-Egger method. The MR-Egger method estimates the effect size by adjusting for horizontal pleiotropy (the genetic variants have effects on the outcome through other paths than via the exposure of interest) (30). Pleiotropy was assessed using the MR-Egger intercept test, which assumes that the intercept should be zero if the genotype–exposure association is zero. To test heterogeneity for the sex-stratified MR analyses with the inverse variance weighted method, we also calculated *P*-heterogeneity by Cochran's Q test.

## Results

By using the linear regression analyses, we obtained summary statistics for the associations between the instrumental variables (genetic variants) and FI that were needed to conduct the MR analysis (Table 1, **Supplemental Tables 1–3**). The analytical sample for genotype–FI associations (*n* = 356,432 participants) comprised 189,949 women (53.3%) and 166,483 men (46.7%), with a mean ± SD age of 56.7 ± 8.0 y and with a mean ± SD FI of 12.0 ± 7.4%.

**FIGURE 1:**
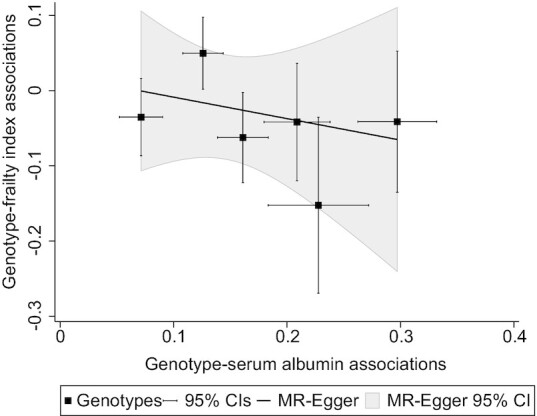
Association between genetically predicted serum albumin (g/L) concentrations and frailty index in women (*n* = 189,949): a result of the MR-Egger method.

The results of the MR analyses of serum albumin are shown in **Table 2**. The results of the inverse variance weighted method in the full sample (both sexes) indicated that a genetically predicted serum albumin concentration was not statistically significantly associated with FI (β per g/L: −0.023; 95% CI: −0.141, 0.094; *P *= 0.694). Results obtained by the weighted median method and MR Egger method were not essentially different from the result based on the inverse variance weighted method. There was no evidence for pleiotropy based on the MR-Egger regression analysis (*P*-intercept = 0.957). However, in women, a genetically-predicted serum albumin concentration was significantly associated with FI (β per g/L: −0.172; 95% CI: −0.336, −0.007; *P *= 0.041). Even when the penalized weights were applied to downweigh the contribution of genetic variants with outlying ratio estimates, a significant result in women was observed (β per g/L: −0.296; 95% CI: −0.477, −0.114; *P *= 0.001). Although point estimates obtained by the weighted median method or MR Egger method were not attenuated compared with the result in the inverse variance weighted method, they were not statistically significant. A plot to visualize the result of the MR Egger method in women is shown in   **Figure 1**. In men, there were no significant associations between genetically predicted serum albumin concentrations and FI in any of the 3 types of MR analyses (Table 2). Statistically significant heterogeneity by sex was observed (*P*-heterogeneity = 0.013).

**TABLE 2:** MR results of the serum albumin and frailty index by using UK Biobank data^1^

MR method	β	(95% CI)	*P* value
All (*n* = 356,432)^2^			
IVW	−0.023	(−0.141, 0.094)	0.694
Penalized IVW	−0.120	(−0.255, 0.016)	0.083
Weighted median	−0.030	(−0.189, 0.129)	0.712
MR-Egger	−0.015	(−0.330, 0.299)	0.923
MR-Egger (intercept)	−0.001		0.957
Women (*n* = 189,949)^2^			
IVW	−0.172	(−0.336, −0.007)	0.041
Penalized IVW	−0.296	(−0.477, −0.114)	0.001
Weighted median	−0.185	(−0.420, 0.050)	0.122
MR-Egger	−0.286	(−0.691, 0.120)	0.167
MR-Egger (intercept)	0.020		0.546
Men (*n* = 166,483)^2^			
IVW	0.123	(−0.041, 0.287)	0.141
Penalized IVW	0.123	(−0.041, 0.287)	0.141
Weighted median	0.150	(−0.050, 0.349)	0.141
MR-Egger	0.217	(−0.232, 0.667)	0.343
MR-Egger (intercept)	−0.017		0.659

1 β, coefficient of serum albumin (g/L); IVW, inverse variance weighted method; MR, Mendelian randomization.

2 Number of participants who were included in the analysis for summary statistics of frailty index.

The MR-Lasso method identified all 6 of the SNPs as valid instruments except for the MR results in women. In the MR results in women, the MR-Lasso method identified only 5 SNPs as valid instruments (identifying rs1260326 as an invalid instrument). The inverse variance weighted method using these 5 valid instruments showed that a genetically predicted serum albumin concentration was significantly associated with FI (β per g/L: −0.302; 95% CI: −0.484, −0.120; *P *= 0.001).

The results of the MR analyses (inverse variance weighted method) of serum total protein are shown in **Table 3**. In the full sample, a genetically predicted serum total protein concentration was significantly associated with FI (β per g/L: −0.153; 95% CI: −0.251, −0.056; *P *= 0.002). In both women and men, higher serum total protein concentrations were significantly inversely associated with FI, and no significant sex difference was observed (*P*-heterogeneity = 0.952).

**TABLE 3:** MR results of the serum total protein and frailty index by using UK Biobank data^1^

	β	(95% CI)	*P* value
All (*n* = 356,432)^2^	−0.153	(−0.251, −0.056)	0.002
Women (*n* = 189,949)^2^	−0.148	(−0.287, −0.009)	0.037
Men (*n* = 166,483)^2^	−0.154	(−0.290, −0.018)	0.027

1 β, coefficient of serum total protein (g/L); MR, Mendelian randomization (inverse variance weighted method).

2 Number of participants who were included in the analysis for summary statistics of frailty index.

We also conducted the following 4 types of sensitivity analyses.

First, we checked the results of the MR analyses using other summary statistics data of protein markers (**Table 4**). These results were not essentially different from the main findings in Table 2. A genetically predicted serum total protein concentration was significantly associated with FI (β per g/L: −0.168; 95% CI: −0.272, −0.064; *P *= 0.002).

**TABLE 4:** Sensitivity analysis: MR results of serum proteins and frailty index using another summary statistics data^1^

MR method	β	(95% CI)	*P* value
Serum albumin			
IVW	−0.046	(−0.160, 0.069)	0.431
Penalized IVW	−0.100	(−0.223, 0.022)	0.109
Weighted median	−0.033	(−0.191, 0.125)	0.683
MR-Egger	−0.207	(−0.864, 0.328)	0.377
MR-Egger (intercept)	0.004		0.443
Serum total protein			
IVW	−0.168	(−0.272, −0.064)	0.002

1 β, coefficient of serum protein markers (g/L); IVW, inverse variance weighted method; MR, Mendelian randomization. The summary statistics are shown in Supplementary Table 3.

Second, we checked the results of the MR analyses (the inverse variance weighted method) using another set of SNPs (**Table 5**, **Supplemental Table 4**). These results were also consistent with the main findings in Table 2. In women, a genetically predicted serum albumin concentration was significantly associated with FI (β per g/L: −0.209; 95% CI: −0.374, −0.044; *P *= 0.013). In addition, a genetically predicted serum total protein concentration was significantly associated with FI in the full sample (β per g/L: −0.133; *P *= 0.002) and in women (β per g/L: −0.176; *P *= 0.004), but not in men.

**TABLE 5:** Sensitivity analysis: MR results of serum proteins and frailty index using another set of SNPs^1^

	β	(95% CI)	*P* value
Serum albumin			
All (*n* = 356,432)^2^	−0.023	(−0.141, 0.094)	0.698
Women (*n* = 189,949)^2^	−0.209	(−0.374, −0.044)	0.013
Men (*n* = 166,483)^2^	0.155	(−0.010, 0.320)	0.066
Serum total protein			
All (*n* = 356,432)^2^	−0.133	(−0.217, −0.050)	0.002
Women (*n* = 189,949)^2^	−0.176	(−0.295, −0.056)	0.004
Men (*n* = 166,483)^2^	−0.088	(−0.204, 0.029)	0.140

1 β, coefficient of serum protein markers (g/L); MR, Mendelian randomization (inverse variance weighted method). Another set of SNPs is shown in Supplemental Table 4.

2 Number of participants who were included in the analysis for summary statistics of frailty index.

Third, we checked the difference in the MR results of serum proteins and FI when stratified by menopausal status (**Table 6**). For both serum albumin and serum total protein, we did not observe statistically significantly results in the inverse variance weighted method regardless of menopausal status among women, and no significant differences by menopausal status were observed in serum albumin (*P*-heterogeneity = 0.811) and serum total protein (*P*-heterogeneity = 0.688).

**TABLE 6:** Stratified analysis by menopausal status: MR results of serum proteins and frailty index by using UK Biobank data (*n* = 160,666 women)^1^

	β	(95% CI)	*P* value
Serum albumin			
Nonmenopausal (*n* = 44,345)^2^	−0.201	(−0.502, 0.099)	0.189
Postmenopausal (*n* = 116,321)^2^	−0.156	(−0.368, 0.055)	0.146
Serum total protein			
Nonmenopausal (*n* = 44,345)^2^	−0.180	(−0.436, 0.076)	0.169
Postmenopausal (*n* = 116,321)^2^	−0.116	(−0.295, 0.062)	0.201

1 β, coefficient of serum protein markers (g/L); MR, Mendelian randomization (inverse variance weighted method).

2 Number of participants who were included in the analysis for summary statistics of frailty index.

Fourth, we checked the difference in the MR results of serum proteins and FI when stratified by age groups (**Supplemental Table 5**). For serum albumin, we did not observe statistically significant results in the inverse variance weighted method regardless age groups, and no significant difference by age was observed (*P*-heterogeneity = 0.921). On the other hand, a genetically predicted serum total protein concentration was significantly associated with FI (β per g/L: −0.275; *P* < 0.001) in the younger age group (<60 y), but not in the older age group (≥60 y), and significant difference by age was observed (*P*-heterogeneity = 0.006).

## Discussion

To the best of our knowledge, this is the first MR study to examine the relation between protein nutritional status and frailty. As a main result, there was no statistically significant association between genetically predicted serum albumin and FI in the full sample. However, in women, we observed an inverse relation between serum albumin concentration and FI. In addition, we observed an inverse relation between genetically predicted serum total protein concentration and FI in the full sample and in both sexes in the sex-stratified analysis. As these results were based only on summary statistics from UK Biobank data (white-British ancestry), they did not impose problems regarding data harmonization in the MR analysis. In addition, even when the summary statistics data of serum proteins were used, the results were not essentially different from the main findings (Table 4).

Although a systematic review of intervention studies has concluded that “a combination of muscle strength training and protein supplementation was the most effective intervention to delay or reverse frailty” (3), investigations of the impact of protein nutritional status alone on frailty have been scarce. The present MR study thus provides evidence to support a causal relation and adds to the understanding of the impact of protein nutritional status alone on frailty.

One of the key methodological issues of MR analysis is the generalizability of genotype–exposure associations. An underestimation of the causal relation by the “winners’ curse” is suggested as a disadvantage of overlapping samples (one-sample MR study), because some statistically significant genotype–exposure associations could be cohort specific (32, 33). However, we selected such SNPs for instrumental variables that have been significantly associated with serum proteins in a previous study (27), and all of these genotype–exposure associations were also replicated in the UK Biobank data (Table 1). Therefore, our MR estimation is unlikely to be affected by the winners’ curse.

Another key methodological issue in MR analysis is biased estimation due to overlapping samples (34). Although the present study is based on a two-sample MR analyses, since the main analysis was based on UK Biobank data for both the exposure and outcome, there is no problem in terms of consistency of the sample characteristics such as ancestry. On the other hand, because the main analysis was based on overlapping samples, biased estimation (e.g., overestimation) may be a concern. However, the main results using the UK Biobank data were not overestimated (Tables 2 and 3) in comparison with the results of the sensitivity analysis based on nonoverlapping samples (Table 4).

We also confirmed the results of our MR analyses using another set of SNPs as instrumental variables that were based on transethnic GWAS meta-analysis of European and Japanese ancestry [SNPs were associated with serum protein concentrations in both European and Japanese ancestry (27)], and these results (Table 5) were consistent with those from the main findings. Most of the SNPs identified in the transethnic GWAS meta-analysis were consistent with those of only European ancestry; 5 SNPs were completely consistent and 3 SNPs (rs739347, rs694419, and rs3751991) were likely to be in high linkage disequilibrium with corresponding SNPs in some chromosomal locations (we checked the D prime value by LDlink and these were >0.92). Only 1 SNP, rs2280401, for total protein concentration was uniquely identified. Therefore, it would be naturally expected that the MR results of serum albumin (Table 5) are consistent with those of the main findings. On the other hand, all of genotype–exposure associations based on the transethnic GWAS meta-analysis (even rs2280401 in total protein) were also replicated in the UK Biobank data, and these findings suggest the reliability of the instrumental variables for serum protein. Therefore, at least the genotype–exposure associations are not limited to European-ancestry, and the results of the present study suggest that the relation between serum proteins and frailty is biologically relevant and plausible.

In addition, MR has the advantage that it is less likely to be affected by confounding and reverse causation than conventional observational studies (24). This advantage would be applicable in the present study if the instrumental variables (SNPs) were not associated with potential confounding traits. In the present study, traits related to lowering serum proteins, such as liver function, cancer, and inflammation (13) should be especially considered. Therefore, we checked each instrumental variable (SNP) and their proxies (*r*^2^ >0.9) in the PhenoScanner GWAS database (version 2; http://phenoscanner.medschl.cam.ac.uk) to assess any previous associations (*P* < 1 × 10^−8^) with these traits. As a result, among 6 serum protein SNPs, rs1260326 was reported for 137 traits, including an inflammatory marker (C-reactive protein) (**Supplemental Table 6**). This SNP was also identified as an invalid instrument by the MR-Lasso method in women. However, even when we removed rs1260326 from the instrumental SNPs in our MR, the result was not essentially different from the main result (β: −0.302; *P *= 0.001). Therefore, the main result in the present study is unlikely to be explained by potential confounding traits.

Interestingly, our results suggest that the inverse associations between serum albumin and FI are more pronounced in women than in men. A previous cross-sectional study has reported that an inverse association between protein intake and prefrailty is seen in women but not in men, although higher protein intake was associated with lower prevalence of frailty in both sexes (35). Because all of the 6 SNPs (instrumental variables) were significantly associated with serum protein markers in both women and men in our study (Supplemental Table 1 and Supplemental Table 2), the difference between sexes is likely not explained by the differences in the genotype–exposure associations. One potential reason may be the differences in the biophysiological mechanisms underlying the association between serum proteins and FI. For example, our FI definition included osteoporosis, fractures, and osteoarthritis, which are more common in women than in men (36). Dietary protein intake is known as a protective factor for these musculoskeletal conditions (36–41). The prevalence of these musculoskeletal conditions is also higher after menopause. Therefore, to consider the possibility that serum proteins are more strongly associated with FI in postmenopausal women, we conducted stratified analyses by menopausal status. However, the relation in postmenopausal women was not more pronounced than in nonmenopausal women (Table 6). Serum albumin is known to have multiple roles in various health outcomes, such as cardiometabolic diseases and cognitive function (12, 16, 42–45). Therefore, the mechanisms underlying the relation between serum proteins and frailty should be understood through not only musculoskeletal conditions but also the other traits. However, because there were significant inverse associations between serum total protein and FI in men (β: −0.154; *P *= 0.027 in Table 3), it would be hard to conclude that serum proteins contribute to frailty prevention only for women based on only results of serum albumin. Further studies are needed to establish sex differences in the association between protein nutritional status and frailty.

The present study has several limitations. First, we only used a relatively small number of SNPs as instrumental variables, especially for serum total protein (only 2 SNPs in the main exposure data). Therefore, we could not conduct MR-Egger regression for serum total protein. Future GWAS studies are thus warranted to explore more SNPs associated with serum protein traits. Second, in the analyses stratified by menopausal status or age, we applied the summary statistics data of genotype–frailty associations by menopausal status or age but not for genotype–exposure associations. These were estimates made under assumptions in which genotype–exposure associations were completely the same regardless of menopausal status or age. Stratified analyses based on one-sample MR analysis would be desirable. Third, because the present analysis was basically restricted to populations of European ancestry, it was unclear that the present findings can be adapted to non-European populations. Fourth, the exposure measures of the present study (serum albumin, serum total protein) are markers of protein nutritional status rather than indicators of dietary protein intake. To directly evaluate the effect of protein supplementation on frailty, clinical trials of protein supplementation would be ultimately necessary.

In conclusion, the results of the present MR study indicate that higher protein nutritional status modestly contributes to reducing the risk of frailty. Diet is a common modifiable factor for most people in daily life. Therefore, even though the preventive effect of maintaining better protein nutritional status on frailty was modest, the public health impact may be considerable in the context of population aging worldwide.

## Supplementary Material

nxab348_Supplemental_File

## Notes

Supported by the Swedish Research Council (grant numbers 2018-02077 and 2019-01272), the Loo & Hans Osterman Foundation, the Strategic Research Program in Epidemiology at Karolinska Institute, and the King Gustaf V and Queen Victoria Freemason Foundation.

Author disclosure: All authors report no conflict of interests.

Supplemental Figure 1 and Supplemental Tables 1–6 are available from the “Supplementary data” link in the online posting of the article and from the same link in the online table of contents at https://academic.oup.com/jn/.

Abbreviations used: FI, frailty index; GWAS, genome-wide association study; MR, Mendelian randomization; RCT, randomized controlled trial.
