# Supplementary material for: Protein Nutritional Status and Frailty: A Mendelian Randomization Study
Source: J Nutr. 2021 Nov 23;152(1):269–75. doi: 10.1093/jn/nxab348 (PMC8754580; doi:10.1093/jn/nxab348)
Supplement: nxab348_Supplemental_File [file nxab348_supplemental_file.docx]

### **Online Supplementary Material.**

### Protein Nutritional Status and Frailty: A Mendelian Randomization Study (Tomata Y, et al.)

**Supplementary Figure 1.** Flowchart of the study participants for the analysis of genotype-frailty index associations.

**Supplementary Table 1.** Summary statistics of serum proteins-raising genetic variants for women ^1^

|  |  | **Effect allele** | **Other allele** | **Serum protein markers** ^2^ | | |  | **Frailty index** ^3^ | | |  |
| --- | --- | --- | --- | --- | --- | --- | --- | --- | --- | --- | --- |
| **SNP** | **Chr** |  |  | ***β*** | **SE** | ***P* value** |  | ***β*** | **SE** | ***P* value** |  |
| **Serum albumin** | | | | | | | | | | |  |
| rs4806073 | 19 | C | T | 0.30 | 0.02 | 1.01×10^-62^ |  | -0.041 | 0.048 | 0.389 |  |
| rs1260326 | 2 | T | C | 0.13 | 0.01 | 1.40×10^-43^ |  | 0.050 | 0.024 | 0.042 |  |
| rs11078597 | 17 | C | T | 0.16 | 0.01 | 2.62×10^-45^ |  | -0.062 | 0.031 | 0.042 |  |
| rs13381710 | 18 | G | A | 0.07 | 0.01 | 2.18×10^-13^ |  | -0.035 | 0.026 | 0.181 |  |
| rs16948098 | 15 | A | G | 0.23 | 0.02 | 7.57×10^-24^ |  | -0.152 | 0.060 | 0.011 |  |
| rs739347 | 19 | T | C | 0.21 | 0.01 | 7.50×10^-45^ |  | -0.042 | 0.040 | 0.295 |  |
|  |  |  |  |  |  |  |  |  |  |  |  |
| **Serum total protein** | | | | | | | | | | |  |
| rs3751991 | 17 | A | C | 0.44 | 0.02 | 1.37×10^-80^ |  | -0.028 | 0.040 | 0.475 |  |
| rs204999 | 6 | A | G | 0.22 | 0.02 | 2.26×10^-50^ |  | -0.064 | 0.026 | 0.014 |  |
| 1. Abbreviations: Chr = chromosome; EAF = effect allele frequency; SE = standard error; SNP = single-nucleotide polymorphism. | | | | | | | | | | | |
| 2. Summary statistics for serum protein markers (albumin, total protein; g/L) from UK Biobank data. | | | | | | | | | | | |
| 3. Summary statistics for frailty index from UK Biobank data. | | | | | | | | | | | |

**Supplementary Table 2.** Summary statistics of serum proteins-raising genetic variants for men ^1^

|  |  | **Effect allele** | **Other allele** | **Serum protein markers** ^2^ | | |  | **Frailty index** ^3^ | | |  |
| --- | --- | --- | --- | --- | --- | --- | --- | --- | --- | --- | --- |
| **SNP** | **Chr** |  |  | ***β*** | **SE** | ***P* value** |  | ***β*** | **SE** | ***P* value** |  |
| **Serum albumin** | | | | | | | | | | |  |
| rs4806073 | 19 | C | T | 0.26 | 0.02 | 4.28×10^-45^ |  | 0.035 | 0.050 | 0.485 |  |
| rs1260326 | 2 | T | C | 0.18 | 0.01 | 1.07×10^-83^ |  | 0.029 | 0.025 | 0.252 |  |
| rs11078597 | 17 | C | T | 0.18 | 0.01 | 2.44×10^-51^ |  | 0.056 | 0.032 | 0.078 |  |
| rs13381710 | 18 | G | A | 0.06 | 0.01 | 3.58×10^-09^ |  | -0.002 | 0.027 | 0.933 |  |
| rs16948098 | 15 | A | G | 0.20 | 0.02 | 2.68×10^-17^ |  | 0.000 | 0.063 | 0.999 |  |
| rs739347 | 19 | T | C | 0.17 | 0.02 | 1.43×10^-26^ |  | -0.043 | 0.041 | 0.304 |  |
|  |  |  |  |  |  |  |  |  |  |  |  |
| **Serum total protein** | | | | | | | | | | |  |
| rs3751991 | 17 | A | C | 0.42 | 0.02 | 5.00×10^-64^ |  | -0.049 | 0.041 | 0.230 |  |
| rs204999 | 6 | A | G | 0.28 | 0.02 | 1.31×10^-67^ |  | -0.052 | 0.027 | 0.054 |  |
| 1. Abbreviations: Chr = chromosome; EAF = effect allele frequency; SE = standard error; SNP = single-nucleotide polymorphism. | | | | | | | | | | | |
| 2. Summary statistics for serum protein markers (albumin, total protein; g/L) from UK Biobank data. | | | | | | | | | | | |
| 3. Summary statistics for frailty index from UK Biobank data. | | | | | | | | | | | |

**Supplementary Table 3.** Summary statistics of serum proteins-raising genetic variants (data for sensitivity analysis) ^1^

|  |  | **Effect allele** | **Other allele** | **Serum protein markers** ^2^ | | | |  | **Frailty index** ^3^ | | |
| --- | --- | --- | --- | --- | --- | --- | --- | --- | --- | --- | --- |
| **SNP** | **Chr** |  |  | **EAF** | ***β*** | **SE** | ***P* value** |  | ***β*** | **SE** | ***P* value** |
| **Serum albumin** | | | | | | | | | | | |
| rs4806073 | 19 | C | T | 0.93 | 0.257 | 0.033 | 3.3×10^-15^ |  | -0.006 | 0.034 | 0.873 |
| rs1260326 | 2 | T | C | 0.41 | 0.124 | 0.016 | 2.9×10^-14^ |  | 0.040 | 0.018 | 0.023 |
| rs11078597 | 17 | C | T | 0.18 | 0.205 | 0.029 | 6.8×10^-13^ |  | -0.007 | 0.022 | 0.757 |
| rs13381710 | 18 | G | A | 0.30 | 0.108 | 0.018 | 3.9×10^-9^ |  | -0.020 | 0.019 | 0.295 |
| rs16948098 | 15 | A | G | 0.06 | 0.229 | 0.041 | 1.9×10^-8^ |  | -0.082 | 0.043 | 0.060 |
| rs739347 | 19 | T | C | 0.89 | 0.186 | 0.034 | 3.2×10^-8^ |  | -0.042 | 0.029 | 0.141 |
|  |  |  |  |  |  |  |  |  |  |  |  |
| **Serum total protein** | | | | | | | | | | | |
| rs3751991 | 17 | A | C | 0.11 | 0.377 | 0.059 | 1.3×10^-10^ |  | -0.038 | 0.029 | 0.182 |
| rs204999 | 6 | A | G | 0.74 | 0.251 | 0.042 | 3.4×10^-9^ |  | -0.058 | 0.019 | 0.002 |
| 1. Abbreviations: Chr = chromosome; EAF = effect allele frequency; SE = standard error; SNP = single-nucleotide polymorphism. | | | | | | | | | | | |
| 2. Summary statistics for serum protein markers (albumin, total protein; g/L) from genome-wide association study of European-ancestry individuals (Franceschini N et al. Am J Hum Genet. 2012;91:744-53). | | | | | | | | | | | |
| 3. Summary statistics for frailty index from UK Biobank data. | | | | | | | | | | | |

**Supplementary Table 4.** Summary statistics of serum proteins-raising genetic variants (another SNPs data for sensitivity analysis) ^1^

|  |  | **Effect allele** | **Other allele** | **Serum protein markers** ^2^ | | | |  | **Frailty index** ^3^ | | |
| --- | --- | --- | --- | --- | --- | --- | --- | --- | --- | --- | --- |
| **SNP** | **Chr** |  |  | **EAF** | ***β*** | **SE** | ***P* value** |  | ***β*** | **SE** | ***P* value** |
| **Serum albumin** | | | | | | | | | | | |
| rs1260326 | 2 | T | C | 0.61 | 0.15 | 0.01 | 5.02×10^-120^ |  | 0.040 | 0.018 | 0.023 |
| rs4806073 | 19 | C | T | 0.07 | 0.28 | 0.01 | 8.08×10^-106^ |  | -0.006 | 0.034 | 0.873 |
| rs11078597 | 17 | C | T | 0.19 | 0.17 | 0.01 | 3.34×10^-94^ |  | -0.007 | 0.022 | 0.757 |
| rs694419 | 18 | T | C | 0.53 | 0.04 | 0.01 | 1.18×10^-09^ |  | -0.036 | 0.017 | 0.035 |
| rs2280401 | 19 | A | G | 0.16 | 0.16 | 0.01 | 1.78×10^-74^ |  | -0.029 | 0.024 | 0.220 |
| rs16948098 | 15 | A | C | 0.04 | 0.21 | 0.02 | 2.02×10^-39^ |  | -0.082 | 0.043 | 0.060 |
|  |  |  |  |  |  |  |  |  |  |  |  |
| **Serum total protein** | | | | | | | | | | | |
| rs4561508 | 17 | T | C | 0.10 | 0.45 | 0.02 | 8.69×10^-155^ |  | -0.032 | 0.029 | 0.263 |
| rs204999 | 6 | A | G | 0.69 | 0.25 | 0.01 | 1.18×10^-114^ |  | -0.058 | 0.019 | 0.002 |
| rs2280401 | 19 | A | G | 0.16 | 0.26 | 0.01 | 3.11×10^-80^ |  | -0.029 | 0.024 | 0.220 |
| 1. Abbreviations: Chr = chromosome; EAF = effect allele frequency; SE = standard error; SNP = single-nucleotide polymorphism. | | | | | | | | | | | |
| 2. Summary statistics for serum protein markers (albumin, total protein; g/L) from UK Biobank data. | | | | | | | | | | | |
| 3. Summary statistics for frailty index from UK Biobank data. | | | | | | | | | | | |

**Supplementary Table 5.** Stratified analysis by age group: MR results of serum proteins and frailty index ^1^

|  | **β** | **(95% CI)** | ***P* value** |
| --- | --- | --- | --- |
| **Serum albumin** | | | |
| **<60 years (n=199,171)** ^2^ | -0.029 | (-0.182, 0.124) | 0.712 |
| **≥60 years (n=157,261)** ^2^ | -0.017 | (-0.197, 0.164) | 0.855 |
|  |  |  |  |
| **Serum total protein** | | | |
| **<60 years (n=199,171)** ^2^ | -0.275 | (-0.403, -0.147) | <0.001 |
| **≥60 years (n=157,261)** ^2^ | 0.002 | (-0.148, 0.152) | 0.978 |
| 1. Abbreviations: MR = Mendelian randomization (inverse variance weighted method); β = coefficient of serum protein markers (g/L); 95% CI = 95% confidence interval.  2. Number of participants who were included in the analysis for summary statistics of frailty index. | | | |

**Supplementary Table 6.** List of the other trait than serum proteins in instrumental SNPs and their proxies obtained by the PhenoScanner.

| **rsid** | **Trait** | **Pubmed ID** | **β** | **SE** | ***P* value** |
| --- | --- | --- | --- | --- | --- |
| rs1260326 | 2 hour glucose | 20081857 | NA | NA | 2.26E-21 |
| rs1260326 | 2 hour glucose tolerance test | 22885924 | NA | NA | 9.04E-15 |
| rs1260326 | Age at menopause | 26414677 | -0.13 | 0.02 | 1.00E-09 |
| rs1260326 | Alcohol consumption | 28937693 | -0.028 | 0.002924 | 1.00E-21 |
| rs1260326 | Alcohol consumption in current drinkers | 28937693 | 0.03 | 0.002977 | 7.00E-24 |
| rs1260326 | Alcohol intake frequency | UKBB | -0.048 | 0.003633 | 7.60E-40 |
| rs1260326 | Alcohol intake versus 10 years previously | UKBB | -0.01242 | 0.001887 | 4.75E-11 |
| rs1260326 | APOA1 assay lipoprotein fraction concentration | 19936222 | NA | NA | 2.87E-11 |
| rs1260326 | APOA1 assay lipoprotein fraction concentration in fasting sample | 19936222 | NA | NA | 3.40E-09 |
| rs1260326 | APOC3 apolipoprotein C III | 19060906 | NA | NA | 8.70E-12 |
| rs1260326 | Arm fat-free mass left | UKBB | 0.01965 | 0.001579 | 1.49E-35 |
| rs1260326 | Arm fat-free mass right | UKBB | 0.02004 | 0.001547 | 2.28E-38 |
| rs1260326 | Arm predicted mass left | UKBB | 0.01948 | 0.001573 | 3.23E-35 |
| rs1260326 | Arm predicted mass right | UKBB | 0.01989 | 0.001542 | 4.46E-38 |
| rs1260326 | Basal metabolic rate | UKBB | 0.02018 | 0.001646 | 1.61E-34 |
| rs1260326 | Blood metabolite levels | 24816252 | -0.044 | 0.002358 | 1.00E-77 |
| rs1260326 | Blood metabolite levels | 24816252 | -0.013 | 0.001731 | 6.00E-14 |
| rs1260326 | Blood metabolite levels | 25898920 | NA | NA | 6.00E-56 |
| rs1260326 | Blood metabolite ratios | 24816252 | -0.041 | 0.001581 | 3.00E-148 |
| rs1260326 | C reactive protein | 21300955 | NA | NA | 5.40E-43 |
| rs1260326 | C reactive protein | 23263486 | NA | NA | 3.80E-43 |
| rs1260326 | C reactive protein | 23505291 | NA | NA | 5.41E-09 |
| rs1260326 | C reactive protein | 21300955 | NA | NA | 5.00E-40 |
| rs1260326 | C reactive protein levels | 21300955 | -0.072 | 0.005437 | 5.00E-40 |
| rs1260326 | Cardiovascular disease risk factors | 21943158 | -0.082 | 0.01461 | 2.00E-08 |
| rs1260326 | Cholesterol total | 24097068 | -0.051 | 0.003744 | 3.00E-42 |
| rs1260326 | Cholesterol total | 25961943 | -0.045 | 0.006169 | 3.00E-13 |
| rs1260326 | Chronic kidney disease | 20383146 | -0.01 | 0.001316 | 3.00E-14 |
| rs1260326 | Comparative height size at age 10 | UKBB | 0.01465 | 0.001696 | 5.72E-18 |
| rs1260326 | Daytime dozing or sleeping | UKBB | -0.007797 | 0.001228 | 2.19E-10 |
| rs1260326 | Diabetes diagnosed by doctor | UKBB | 0.003817 | 0.0005326 | 7.62E-13 |
| rs1260326 | DPA 22:5n3 | 21829377 | -0.0165 | 0.0029 | 1.44E-08 |
| rs1260326 | Fasting blood glucose | 20081857 | NA | NA | 4.30E-13 |
| rs1260326 | Fasting blood glucose | 20081858 | NA | NA | 4.25E-13 |
| rs1260326 | Fasting blood glucose | 22885924 | NA | NA | 2.17E-41 |
| rs1260326 | Fasting blood glucose | 23263486 | NA | NA | 4.30E-13 |
| rs1260326 | Fasting glucose | 22885924 | 0.029 | 0.0021 | 2.17E-41 |
| rs1260326 | Fasting glucose | 20081858 | 0.027 | 0.0037 | 4.25E-13 |
| rs1260326 | Fasting insulin | 22885924 | NA | NA | 2.74E-22 |
| rs1260326 | FVII in plasma | 20231535 | NA | NA | 6.20E-24 |
| rs1260326 | Glomerular filtration rate creatinine | 26831199 | -0.0068 | 0.0008949 | 3.00E-14 |
| rs1260326 | Glomerular filtration rate in non diabetics creatinine | 26831199 | -0.0065 | 0.000924 | 2.00E-12 |
| rs1260326 | Glucose tolerance test | 20081857 | NA | NA | 3.00E-10 |
| rs1260326 | Granulocyte count | 27863252 | -0.03151 | 0.003656 | 6.81E-18 |
| rs1260326 | Granulocyte count | 27863252 | 0.03151 | 0.003657 | 7.00E-18 |
| rs1260326 | Granulocyte percentage of myeloid white cells | 27863252 | -0.03934 | 0.003636 | 2.81E-27 |
| rs1260326 | HDL cholesterol mean size lipoprotein fraction concentration | 19936222 | NA | NA | 7.20E-10 |
| rs1260326 | HDL cholesterol small lipoprotein fraction concentration | 19936222 | NA | NA | 8.39E-21 |
| rs1260326 | HDL cholesterol total lipoprotein fraction concentration | 19936222 | NA | NA | 6.30E-36 |
| rs1260326 | HDL cholesterol total lipoprotein fraction concentration in fasting sample | 19936222 | NA | NA | 8.00E-28 |
| rs1260326 | Height | 25282103 | 0.02 | 0.003 | 1.40E-11 |
| rs1260326 | Height | 28146470 | 0.017 | 0.0027 | 8.40E-11 |
| rs1260326 | Height | UKBB | 0.01674 | 0.001765 | 2.48E-21 |
| rs1260326 | Hematocrit | 27863252 | 0.02209 | 0.003594 | 7.85E-10 |
| rs1260326 | Hematocrit | 27863252 | 0.02209 | 0.003595 | 8.00E-10 |
| rs1260326 | High light scatter percentage of red cells | 27863252 | -0.03532 | 0.00365 | 3.71E-22 |
| rs1260326 | High light scatter reticulocyte count | 27863252 | -0.03219 | 0.00365 | 1.15E-18 |
| rs1260326 | High light scatter reticulocyte percentage of red cells | 27863252 | 0.03532 | 0.003653 | 4.00E-22 |
| rs1260326 | Hypertriglyceridemia | 20657596 | NA | NA | 6.50E-09 |
| rs1260326 | Hypertriglyceridemia | 20657596 | -0.5596 | 0.09664 | 7.00E-09 |
| rs1260326 | Hypertriglyceridemia | 20657596 | NA | NA | 7.00E-09 |
| rs1260326 | IFT172 expression in Lymphocytes lymphoblastoid cell lines tissue | 20383146 | NA | NA | 7.00E-12 |
| rs1260326 | Impedance of arm left | UKBB | -0.01918 | 0.001754 | 7.79E-28 |
| rs1260326 | Impedance of arm right | UKBB | -0.01881 | 0.001746 | 4.81E-27 |
| rs1260326 | Impedance of leg left | UKBB | -0.0211 | 0.002264 | 1.19E-20 |
| rs1260326 | Impedance of leg right | UKBB | -0.02049 | 0.002247 | 7.69E-20 |
| rs1260326 | Impedance of whole body | UKBB | -0.02163 | 0.001893 | 3.21E-30 |
| rs1260326 | Inflammatory bowel disease | 26192919 | -0.07711 | 0.009964 | 1.00E-14 |
| rs1260326 | Kidney diseases | 20383146 | NA | NA | 3.00E-14 |
| rs1260326 | Leg fat-free mass left | UKBB | 0.01842 | 0.001635 | 2.01E-29 |
| rs1260326 | Leg fat-free mass right | UKBB | 0.01843 | 0.001634 | 1.73E-29 |
| rs1260326 | Leg predicted mass left | UKBB | 0.01824 | 0.001624 | 2.83E-29 |
| rs1260326 | Leg predicted mass right | UKBB | 0.01839 | 0.001624 | 1.03E-29 |
| rs1260326 | Lipid metabolism phenotypes | 19936222 | NA | NA | 3.00E-29 |
| rs1260326 | Lipid metabolism phenotypes | 19936222 | NA | NA | 4.00E-24 |
| rs1260326 | Lipid metabolism phenotypes | 19936222 | NA | NA | 3.00E-28 |
| rs1260326 | Lipid metabolism phenotypes | 19936222 | NA | NA | 4.00E-32 |
| rs1260326 | Lipid metabolism phenotypes | 19936222 | NA | NA | 3.00E-35 |
| rs1260326 | Lipid metabolism phenotypes | 19936222 | NA | NA | 1.00E-37 |
| rs1260326 | Lipoprotein associated phospholipase A2 mass Lp2 | 23118302 | NA | NA | 9.40E-09 |
| rs1260326 | log eGFR creatinine | 20383146 | NA | NA | 1.30E-10 |
| rs1260326 | log eGFR creatinine | 26831199 | -0.0068 | 0.00092 | 3.40E-14 |
| rs1260326 | log eGFR creatinine in non diabetics | 26831199 | -0.0065 | 0.00092 | 1.90E-12 |
| rs1260326 | log Fasting insulin | 22885924 | 0.019 | 0.0026 | 3.84E-14 |
| rs1260326 | log Fasting insulin adjusted for BMI | 22885924 | 0.021 | 0.0021 | 2.74E-22 |
| rs1260326 | Lymphocyte count | 27863252 | -0.02574 | 0.003664 | 2.11E-12 |
| rs1260326 | Lymphocyte counts | 27863252 | 0.02574 | 0.00366 | 2.00E-12 |
| rs1260326 | Medication for cholesterol, blood pressure or diabetes: cholesterol lowering medication | UKBB | -0.01216 | 0.001551 | 4.58E-15 |
| rs1260326 | Metabolic traits | 19060910 | -0.09 | 0.01439 | 4.00E-10 |
| rs1260326 | Metabolism | 22286219 | NA | NA | 3.00E-18 |
| rs1260326 | Metabolite levels | 22286219 | NA | NA | 3.00E-18 |
| rs1260326 | Metabolite levels | 22916037 | NA | NA | 1.00E-12 |
| rs1260326 | Metabolite levels small molecules and protein measures | 27005778 | 0.1 | 0.009506 | 7.00E-26 |
| rs1260326 | Monocyte percentage of white cells | 27863252 | 0.04419 | 0.003624 | 3.37E-34 |
| rs1260326 | Myeloid white cell count | 27863252 | -0.02869 | 0.003665 | 4.95E-15 |
| rs1260326 | Myeloid white cell count | 27863252 | 0.02869 | 0.003666 | 5.00E-15 |
| rs1260326 | Neutrophil count | 27863252 | -0.03308 | 0.003646 | 1.16E-19 |
| rs1260326 | Neutrophil count | 27863252 | 0.03308 | 0.00364 | 1.00E-19 |
| rs1260326 | Number of self-reported non-cancer illnesses | UKBB | -0.01122 | 0.002052 | 4.53E-08 |
| rs1260326 | Palmitoleic acid 16:1n7 | 23362303 | -0.0195 | 0.0033 | 3.75E-09 |
| rs1260326 | Plasma C reactive protein female | 18439548 | NA | NA | 3.57E-14 |
| rs1260326 | Plasma docosapentaenoic acid levels | 21829377 | NA | NA | 1.44E-08 |
| rs1260326 | Plasma palmitoleic acid | 23362303 | NA | NA | 3.75E-09 |
| rs1260326 | Plasma protein C levels | 20802025 | NA | NA | 2.04E-17 |
| rs1260326 | Platelet count | 27863252 | -0.0386 | 0.00371 | 2.31E-25 |
| rs1260326 | Platelet count | 27863252 | 0.0386 | 0.003705 | 2.00E-25 |
| rs1260326 | Platelet count PLT | 22139419 | NA | NA | 9.12E-10 |
| rs1260326 | Platelet counts | 22139419 | -2.334 | 0.381 | 9.00E-10 |
| rs1260326 | Plateletcrit | 27863252 | -0.0356 | 0.003723 | 1.15E-21 |
| rs1260326 | Plateletcrit | 27863252 | 0.0356 | 0.003718 | 1.00E-21 |
| rs1260326 | Pulse rate | UKBB | -0.02788 | 0.002559 | 1.23E-27 |
| rs1260326 | Pulse rate | UKBB | -0.02641 | 0.004339 | 1.15E-09 |
| rs1260326 | Red blood cell count | 27863252 | 0.02065 | 0.003617 | 1.15E-08 |
| rs1260326 | Red cell distribution width | 27863252 | 0.02457 | 0.003604 | 9.38E-12 |
| rs1260326 | Red cell distribution width | 27863252 | 0.02457 | 0.003601 | 9.00E-12 |
| rs1260326 | Relative age of first facial hair | UKBB | -0.008943 | 0.00162 | 3.41E-08 |
| rs1260326 | Reticulocyte count | 27863252 | -0.03311 | 0.003656 | 1.36E-19 |
| rs1260326 | Reticulocyte fraction of red cells | 27863252 | -0.03866 | 0.003654 | 3.68E-26 |
| rs1260326 | Self-reported diabetes | UKBB | 0.003066 | 0.0004829 | 2.16E-10 |
| rs1260326 | Self-reported gout | UKBB | -0.00323 | 0.0002936 | 3.83E-28 |
| rs1260326 | Self-reported high cholesterol | UKBB | -0.01023 | 0.000814 | 3.17E-36 |
| rs1260326 | Serum creatinine | 20383146 | NA | NA | 3.40E-12 |
| rs1260326 | Serum creatinine estimated glomerular filtration rate eGFR | 20383146 | NA | NA | 3.00E-14 |
| rs1260326 | Serum creatinine estimated glomerular filtration rate eGFR | 22479191 | NA | NA | 4.48E-12 |
| rs1260326 | Serum creatinine estimated glomerular filtration rate eGFR age 65 | 22479191 | NA | NA | 4.30E-14 |
| rs1260326 | Serum creatinine estimated glomerular filtration rate eGFR females | 22479191 | NA | NA | 3.80E-12 |
| rs1260326 | Serum creatinine estimated glomerular filtration rate eGFR no diabetes | 20383146 | NA | NA | 3.80E-10 |
| rs1260326 | Serum creatinine estimated glomerular filtration rate eGFR no hypertension | 20383146 | NA | NA | 6.20E-09 |
| rs1260326 | Serum creatinine estimated glomerular filtration rate eGFR without diabetes | 22479191 | NA | NA | 7.70E-11 |
| rs1260326 | Serum creatinine estimated glomerular filtration rate eGFR without hypertension | 22479191 | NA | NA | 9.70E-10 |
| rs1260326 | Serum urate | 20884846 | NA | NA | 5.90E-17 |
| rs1260326 | Serum urate | 23263486 | NA | NA | 1.25E-44 |
| rs1260326 | Serum urate | 23263486 | -0.077 | 0.0055 | 1.31E-40 |
| rs1260326 | Sitting height | UKBB | 0.01941 | 0.00192 | 5.04E-24 |
| rs1260326 | Sodium in urine | UKBB | -0.01953 | 0.002418 | 6.68E-16 |
| rs1260326 | Sum basophil neutrophil counts | 27863252 | -0.03252 | 0.003653 | 5.49E-19 |
| rs1260326 | Sum basophil neutrophil counts | 27863252 | 0.03252 | 0.003649 | 5.00E-19 |
| rs1260326 | Sum neutrophil eosinophil counts | 27863252 | -0.03203 | 0.00365 | 1.70E-18 |
| rs1260326 | Sum neutrophil eosinophil counts | 27863252 | 0.03203 | 0.003657 | 2.00E-18 |
| rs1260326 | Total cholesterol | 24097068 | -0.0512 | 0.0036 | 3.08E-42 |
| rs1260326 | Total cholesterol | 20339536 | NA | NA | 4.99E-15 |
| rs1260326 | Total cholesterol | 23063622 | NA | NA | 2.33E-19 |
| rs1260326 | Treatment with allopurinol | UKBB | -0.002605 | 0.0002624 | 3.24E-23 |
| rs1260326 | Treatment with atorvastatin | UKBB | -0.00323 | 0.0004254 | 3.15E-14 |
| rs1260326 | Treatment with cholesterol lowering medication | UKBB | -0.008473 | 0.001132 | 7.06E-14 |
| rs1260326 | Treatment with simvastatin | UKBB | -0.004792 | 0.0007876 | 1.17E-09 |
| rs1260326 | Triglycerides | 24097068 | -0.1148 | 0.0034 | 2.29E-239 |
| rs1260326 | Triglycerides | 18193043 | NA | NA | 5.60E-22 |
| rs1260326 | Triglycerides | 19060906 | NA | NA | 1.66E-33 |
| rs1260326 | Triglycerides | 19060911 | NA | NA | 5.17E-17 |
| rs1260326 | Triglycerides | 21943158 | NA | NA | 2.00E-08 |
| rs1260326 | Triglycerides | 23063622 | NA | NA | 4.05E-88 |
| rs1260326 | Triglycerides | 19060906 | -0.12 | 0.01029 | 2.00E-31 |
| rs1260326 | Triglycerides | 24097068 | -0.115 | 0.00348 | 2.00E-239 |
| rs1260326 | Triglycerides | 25961943 | -0.123 | 0.006184 | 5.00E-88 |
| rs1260326 | Triglycerides | 19060906 | NA | NA | 2.00E-31 |
| rs1260326 | Triglycerides | 19060910 | NA | NA | 3.56E-10 |
| rs1260326 | Triglycerides | 19060910 | NA | NA | 4.00E-10 |
| rs1260326 | Triglycerides | 21943158 | NA | NA | 2.00E-08 |
| rs1260326 | Triglycerides by NMR lipoprotein fraction | 19936222 | NA | NA | 2.90E-35 |
| rs1260326 | Triglycerides by NMR lipoprotein fraction in fasting sample | 19936222 | NA | NA | 3.48E-29 |
| rs1260326 | Triglycerides change with statins | 20339536 | NA | NA | 5.00E-15 |
| rs1260326 | Triglycerides in females | 23063622 | NA | NA | 3.90E-38 |
| rs1260326 | Triglycerides in males | 23063622 | NA | NA | 1.17E-42 |
| rs1260326 | Triglycerides lipoprotein fraction | 19936222 | NA | NA | 1.00E-37 |
| rs1260326 | Triglycerides lipoprotein fraction in fasting sample | 19936222 | NA | NA | 4.45E-32 |
| rs1260326 | Triglycerides ln | 19802338 | NA | NA | 1.30E-16 |
| rs1260326 | Triglycerides ln | 23063622 | NA | NA | 5.08E-98 |
| rs1260326 | Triglycerides mmoll | 19060910 | NA | NA | 5.18E-11 |
| rs1260326 | Trunk fat-free mass | UKBB | 0.02196 | 0.001564 | 9.39E-45 |
| rs1260326 | Trunk predicted mass | UKBB | 0.02192 | 0.001559 | 6.87E-45 |
| rs1260326 | Two hour glucose challenge | 20081857 | -0.07 | 0.01111 | 3.00E-10 |
| rs1260326 | Type II diabetes | 26551672 | 0.07696 | 0.01299 | 3.70E-09 |
| rs1260326 | Urate levels | 23263486 | -0.074 | 0.005274 | 1.00E-44 |
| rs1260326 | Uric acid | 19503597 | NA | NA | 1.82E-09 |
| rs1260326 | VLDL cholesterol large lipoprotein fraction concentration | 19936222 | NA | NA | 2.79E-28 |
| rs1260326 | VLDL cholesterol large lipoprotein fraction concentration in fasting sample | 19936222 | NA | NA | 3.60E-24 |
| rs1260326 | VLDL cholesterol mean size lipoprotein fraction concentration | 19936222 | NA | NA | 7.60E-16 |
| rs1260326 | VLDL cholesterol mean size lipoprotein fraction concentration in fasting sample | 19936222 | NA | NA | 6.20E-14 |
| rs1260326 | VLDL cholesterol medium lipoprotein fraction concentration | 19936222 | NA | NA | 3.67E-17 |
| rs1260326 | VLDL cholesterol medium lipoprotein fraction concentration in fasting sample | 19936222 | NA | NA | 6.10E-15 |
| rs1260326 | VLDL cholesterol total lipoprotein fraction concentration | 19936222 | NA | NA | 7.78E-17 |
| rs1260326 | Weight | UKBB | 0.01778 | 0.00217 | 2.53E-16 |
| rs1260326 | White blood cell count | 27863252 | -0.03411 | 0.003648 | 8.75E-21 |
| rs1260326 | White blood cell count | 27863252 | 0.03411 | 0.003649 | 9.00E-21 |
| rs1260326 | Whole body fat-free mass | UKBB | 0.02108 | 0.00157 | 4.40E-41 |
| rs1260326 | Whole body water mass | UKBB | 0.02132 | 0.001572 | 7.14E-42 |
|  |  |  |  |  |  |
| rs16948098 | Hemoglobin concentration | 27863252 | -0.05045 | 0.008913 | 1.51E-08 |
| rs16948098 | High density lipoprotein | 24097068 | -0.0663 | 0.009 | 2.01E-12 |
| rs16948098 | Platelet count | 27863252 | 0.05831 | 0.009167 | 2.01E-10 |
| rs16948098 | Plateletcrit | 27863252 | 0.06232 | 0.009199 | 1.25E-11 |
| rs16948098 | Triglycerides | 24097068 | 0.08 | 0.0089 | 4.84E-17 |
|  |  |  |  |  |  |
| rs204999 | Adolescentyoung adult nodular sclerosis Hodgkin lymphoma | 22086417 | NA | NA | 1.44E-09 |
| rs204999 | Ag×10-related macular degeneration | 26691988 | NA | NA | 1.37E-11 |
| rs204999 | Arm fat-free mass left | UKBB | -0.01437 | 0.001671 | 8.07E-18 |
| rs204999 | Arm fat-free mass right | UKBB | -0.01456 | 0.001638 | 5.98E-19 |
| rs204999 | Arm predicted mass left | UKBB | -0.01448 | 0.001665 | 3.37E-18 |
| rs204999 | Arm predicted mass right | UKBB | -0.01468 | 0.001632 | 2.33E-19 |
| rs204999 | Asthma | UKBB | -0.005033 | 0.000842 | 2.27E-09 |
| rs204999 | Basal metabolic rate | UKBB | -0.01576 | 0.001743 | 1.53E-19 |
| rs204999 | Doctor diagnosed sarcoidosis | UKBB | -0.002049 | 0.0003418 | 2.05E-09 |
| rs204999 | Eosinophil count | 27863252 | 0.02921 | 0.003847 | 3.15E-14 |
| rs204999 | Forced expiratory volume in 1-second, predicted percentage | UKBB | 0.02795 | 0.004589 | 1.13E-09 |
| rs204999 | Granulocyte count | 27863252 | 0.02385 | 0.003874 | 7.37E-10 |
| rs204999 | Hearing difficulty or problems with background noise | UKBB | 0.007218 | 0.001282 | 1.81E-08 |
| rs204999 | Height | UKBB | -0.01434 | 0.001867 | 1.63E-14 |
| rs204999 | Hip circumference | UKBB | -0.01741 | 0.002602 | 2.25E-11 |
| rs204999 | Idiopathic membranous nephropathy | 21323541 | NA | NA | 9.01E-40 |
| rs204999 | Idiopathic membranous nephropathy | 21323541 | NA | NA | 9.00E-40 |
| rs204999 | IgA deficiency | 27723758 | -1 | 0.04916 | 4.48E-92 |
| rs204999 | Impedance of arm left | UKBB | 0.0105 | 0.001856 | 1.52E-08 |
| rs204999 | Impedance of whole body | UKBB | 0.0122 | 0.002004 | 1.15E-09 |
| rs204999 | Intestinal malabsorption | UKBB | -0.002595 | 0.0001133 | 4.45E-116 |
| rs204999 | Leg fat-free mass left | UKBB | -0.0145 | 0.00173 | 5.33E-17 |
| rs204999 | Leg fat-free mass right | UKBB | -0.01457 | 0.00173 | 3.64E-17 |
| rs204999 | Leg predicted mass left | UKBB | -0.01438 | 0.001719 | 5.88E-17 |
| rs204999 | Leg predicted mass right | UKBB | -0.01427 | 0.001719 | 1.02E-16 |
| rs204999 | Lymphocyte count | 27863252 | 0.0286 | 0.003883 | 1.79E-13 |
| rs204999 | Mean corpuscular volume | 27863252 | -0.02142 | 0.003797 | 1.68E-08 |
| rs204999 | Mean platelet volume | 27863252 | -0.03083 | 0.003906 | 2.95E-15 |
| rs204999 | Medication for cholesterol, blood pressure or diabetes: insulin | UKBB | -0.002514 | 0.000453 | 2.87E-08 |
| rs204999 | Monocyte count | 27863252 | 0.04054 | 0.003853 | 6.91E-26 |
| rs204999 | Mouth or teeth dental problems: dentures | UKBB | -0.005547 | 0.000988 | 1.97E-08 |
| rs204999 | Myeloid white cell count | 27863252 | 0.02742 | 0.003883 | 1.66E-12 |
| rs204999 | Neutrophil count | 27863252 | 0.02144 | 0.003864 | 2.85E-08 |
| rs204999 | Nodular sclerosis Hodgkin lymphoma | 22086417 | NA | NA | 8.00E-18 |
| rs204999 | Other rheumatoid arthritis | UKBB | 0.0007738 | 0.0001408 | 3.86E-08 |
| rs204999 | Peak expiratory flow | UKBB | 0.01361 | 0.002338 | 5.83E-09 |
| rs204999 | Platelet count | 27863252 | 0.03805 | 0.003934 | 3.94E-22 |
| rs204999 | Plateletcrit | 27863252 | 0.028 | 0.003944 | 1.26E-12 |
| rs204999 | Primary sclerosing cholangitis | 27992413 | -0.437 | 0.031 | 1.07E-47 |
| rs204999 | Red blood cell count | 27863252 | 0.02644 | 0.003831 | 5.09E-12 |
| rs204999 | Reticulocyte count | 27863252 | 0.02652 | 0.003874 | 7.70E-12 |
| rs204999 | Rheumatoid arthritis | 17804836 | NA | NA | 3.53E-29 |
| rs204999 | Rheumatoid arthritis | 20453842 | NA | NA | 2.50E-63 |
| rs204999 | Rheumatoid arthritis | 24390342 | 0.4253 | 0.01829 | 5.20E-113 |
| rs204999 | Rheumatoid arthritis | 20453842 | 0.462 | 0.02431 | 2.50E-63 |
| rs204999 | Rheumatoid arthritis ACPA positive | 21156761 | NA | NA | 6.62E-24 |
| rs204999 | Self-reported ankylosing spondylitis | UKBB | 0.001129 | 0.0001411 | 1.25E-15 |
| rs204999 | Self-reported asthma | UKBB | -0.004687 | 0.0008436 | 2.76E-08 |
| rs204999 | Self-reported eczema or dermatitis | UKBB | 0.002502 | 0.0004185 | 2.27E-09 |
| rs204999 | Self-reported hyperthyroidism or thyrotoxicosis | UKBB | -0.001799 | 0.0002281 | 3.04E-15 |
| rs204999 | Self-reported malabsorption or coeliac disease | UKBB | -0.006776 | 0.0001723 | 0 |
| rs204999 | Self-reported psoriasis | UKBB | -0.003161 | 0.0002809 | 2.22E-29 |
| rs204999 | Self-reported rheumatoid arthritis | UKBB | 0.001623 | 0.0002757 | 3.92E-09 |
| rs204999 | Sitting height | UKBB | -0.0189 | 0.002031 | 1.38E-20 |
| rs204999 | Started insulin within one year diagnosis of diabetes | UKBB | -0.03063 | 0.003981 | 1.54E-14 |
| rs204999 | Sum basophil neutrophil counts | 27863252 | 0.02198 | 0.00387 | 1.34E-08 |
| rs204999 | Sum eosinophil basophil counts | 27863252 | 0.02984 | 0.003851 | 9.38E-15 |
| rs204999 | Sum neutrophil eosinophil counts | 27863252 | 0.02346 | 0.003867 | 1.30E-09 |
| rs204999 | Systemic sclerosis | 20383147 | NA | NA | 2.30E-09 |
| rs204999 | Treatment with insulin | UKBB | -0.002058 | 0.0003137 | 5.38E-11 |
| rs204999 | Treatment with insulin product | UKBB | -0.00225 | 0.0002603 | 5.41E-18 |
| rs204999 | Trunk fat-free mass | UKBB | -0.01546 | 0.001655 | 9.48E-21 |
| rs204999 | Trunk predicted mass | UKBB | -0.0153 | 0.00165 | 1.77E-20 |
| rs204999 | Type 1 diabetes | 17632545 | NA | NA | 1.90E-09 |
| rs204999 | Ulcerative colitis | 23128233 | NA | NA | 1.21E-11 |
| rs204999 | Weight | UKBB | -0.01839 | 0.002296 | 1.18E-15 |
| rs204999 | White blood cell count | 27863252 | 0.03548 | 0.003866 | 4.47E-20 |
| rs204999 | Whole body fat-free mass | UKBB | -0.01545 | 0.001662 | 1.45E-20 |
| rs204999 | Whole body water mass | UKBB | -0.01522 | 0.001664 | 6.02E-20 |
